# Reduced WNT5A signaling in melanoma cells favors an amoeboid mode of invasion

**DOI:** 10.1002/1878-0261.12974

**Published:** 2021-05-15

**Authors:** Njainday Pulo Jobe, Lisa Åsberg, Tommy Andersson

**Affiliations:** ^1^ Experimental Pathology Department of Translational Medicine Skåne University Hospital Lund University Malmö Sweden

**Keywords:** amoeboid, melanoma invasion, rho GTPase, WNT5A signaling

## Abstract

Tumor cells invade and spread via either a mesenchymal or an amoeboid mode of migration. Amoeboid tumor cells have a rounded morphology and pronounced RhoA activity. Here, we investigate how WNT5A signaling, a tumor promotor in melanoma, relates to Rho GTPase activity and amoeboid migration. We compared melanoma cells with low (HTB63 cells) and high (WM852 cells) WNT5A expression. HTB63 cells exhibited an amoeboid morphology and had higher RhoA activity but lower invasiveness than WM852 cells in a three‐dimensional (3D) collagen matrix. We next explored the relationships between WNT5A, morphology, and invasive behavior. WNT5A knockdown impaired Rho GTPase Cdc42 activity, resulting in reduced invasion of amoeboid and mesenchymal melanoma cells. Interestingly, knockdown of WNT5A or inhibition of its secretion in WM852 cells expressing wild‐type BRAF also led to increased RhoA activity via decreased RND3 expression, resulting in predominantly amoeboid morphology. In contrast, such treatments had the opposite effects on RND3 expression and RhoA activity in HTB63 cells expressing the active BRAF^V600^ mutation. However, treatment of HTB63 cells with a BRAF inhibitor made them respond to WNT5A knockdown in a similar manner as WM852 cells expressing wild‐type BRAF. We next found that dual targeting of WNT5A and RhoA more effectively reduced melanoma cell invasion than targeting either protein individually. Taken together, our results suggest that low WNT5A signaling in melanoma cells promotes a rounded amoeboid type of invasion, which quite likely serves as a compensatory response to decreased WNT5A/Cdc42‐driven invasion. This phenomenon partially explains the enduring melanoma cell invasion observed after impaired WNT5A signaling and has therapeutic implications. Our results suggest that dual targeting of WNT5A and RhoA signaling is a more effective strategy for controlling the invasion of BRAF wild‐type and BRAF^V600^ mutated melanomas treated with a BRAF inhibitor than targeting either of the proteins individually.

## Graphical abstract

Depletion of WNT5a inhibits melanoma cell invasion. Here, we show that WNT5a inhibition is coupled to increased RhoA activity in both BRAF wild‐type and BRAF^V600E^‐mutated cells treated with a BRAF inhibitor. RND3, an atypical RhoA GTPase, counteracted RhoA activity and regulated amoeboid type of invasion. In conclusion, dual WNT5A and RhoA targeting could offer a more effective strategy for blocking melanoma cell invasion.

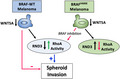

**Abbreviations**
3D: three dimensional
ECM: extracellular matrix
FBS: fetal bovine serum
FZD: Frizzled
GEF: guanine exchange factor
MCAM: melanoma cell adhesion molecule
MMP: matrix metalloprotease
rWNT5A: recombinant WNT5A

## 1 Introduction

Cell migration is a complex process that allows individual cells or cell collectives to move, become displaced, and contribute to the maintenance and/or formation of tissues and organs, development, homeostasis, and regeneration [1]. During cancer progression, tumor cells gain an increased ability to migrate in a similar manner, leading to the spreading of cancer cells to near or distant sites and thus providing a basis for the formation of metastases [2]. Cancer cell migration takes place via a dynamic multistep mechanism that integrates molecular and physical processes, such as cell polarity, cell–cell and cell–matrix adhesion, and proteolysis [3, 4]. There are numerous mechanisms underlying the invasion of tumors, including multicellular, collective, and/or individual mesenchymal or amoeboid migration [3, 5]. Cancer cells can transition between various modes of migration due to various changes within the cell and in the surrounding tumor microenvironment [6]. Cancer cells can undergo amoeboid‐to‐mesenchymal or mesenchymal‐to‐amoeboid transition as a compensatory mechanism. Such transitions are generally mediated and regulated by ligand‐induced activation of different intracellular signaling events to maintain and optimize the invasive capabilities of cancer cells.

Malignant melanoma is an aggressive form of skin cancer and, as such, is characterized by elevated migration and invasion of melanoma cells. *In vitro* studies have shown that the ligand WNT5A plays an essential role in regulating and promoting the invasive migration of melanoma cells [7, 8, 9, 10]. The clinical importance of WNT5A is highlighted by previous results revealing increased expression of WNT5A at both the mRNA and protein levels in clinical melanoma samples compared to normal samples, suggesting that WNT5A promotes melanoma progression [11, 12]. This notion is further supported by the finding that WNT5A expression levels are positively correlated with poor outcomes and poor patient survival [9, 12].

WNT5A is a glycosylated and lipid‐modified secreted protein ligand that predominantly triggers the activation of noncanonical WNT signaling pathways. These WNT5A signaling events are activated upon binding of the ligand to various cell surface receptors, such as members of the Frizzled (FZD) family of receptors and receptor tyrosine kinases, such as ROR2 and RYK [13]. WNT5A signaling pathways have been shown to be involved in numerous biological processes, such as embryonic development [14], tissue regeneration, and wound healing [15]. Aberrant WNT5A expression has been implicated not only in melanoma progression but also in the progression of a number of other cancers. The immediate events following an interaction between the WNT5A ligand and its receptor(s) and which then transduces further downstream signaling including regulation of small GTPases are not completely clear. There are convincing data published on the ability of WNT proteins, including the WNT5A ligand, to initiate associations between the transducing Dishevelled protein and tyrosine kinase as well as Frizzled receptors [16, 17]. In addition, binding of WNT5A to seven‐transmembrane Frizzled receptors has also revealed that heterotrimeric G proteins can serve as transducers of further downstream signaling [17]. It is reasonable to assume that the ability of the WNT5A ligand to bind to different receptors and the presence of two different immediate transducers indicates that its downstream signaling can be both cellular and context dependent. The possibility that the transducers have different time kinetics [17] might indicate that the two transducers complement each other in generating a complete WNT5A‐induced cellular response.

Upon receptor binding, WNT5A activates several intracellular downstream signals, such as Ca^2+^, JNK, and small Rho GTPases [18]. In melanoma, a number of these downstream signaling pathways have been shown to be involved in WNT5A‐dependent cell migration and invasion [7, 9, 19, 20].

Previous studies have emphasized an essential role for WNT5A in the regulation of melanoma cell invasion by, for example, inhibiting WNT5A signaling with the small peptide inhibitor Box5 [7, 8, 21]. However, during such inhibition of WNT5A signaling, some melanoma cells maintain their invasion ability [8]. It is possible that this phenomenon could be related to an ability of WNT5A to regulate melanoma cell plasticity, although this hypothesis has not been completely studied. However, what has been shown is that WNT5A promotes epithelial‐to‐mesenchymal transition of melanoma cells [10] and that it triggers an increase in matrix metalloprotease (MMP) activity and a decrease E‐cadherin expression in epithelial carcinoma [22], implicating a potential role of the WNT5A ligand in tumor cell plasticity. Clearly, whether and how WNT5A promotes the mesenchymal‐to‐amoeboid and amoeboid‐to‐mesenchymal transitions of melanoma cells and thus their invasiveness and metastatic potential are not clearly defined or understood.

In the present study, we analyzed how WNT5A expression and signaling relate to phenotypic changes and the invasiveness of melanoma cells. We found that reduced WNT5A signaling significantly inhibits Cdc42 activity and melanoma cell invasion in a spheroid invasion assay. However, it can also trigger increased RhoA signaling by reducing the expression of RND3/RhoE, a negative regulator of RhoA signaling. These changes lead to a compensatory mesenchymal‐to‐amoeboid transition that counteracts the decreased Cdc42‐driven invasion. These data indicate that simultaneous inhibition of WNT5A and RhoA signaling is a more effective strategy for hindering tumor cell invasion of BRAF wild‐type and BRAF^V600^ mutated melanomas treated with a BRAF inhibitor than targeting either WNT5A or RhoA individually.

## 2 Materials and methods

### 2.1 Cell lines and culture conditions

A2058 (Cat# ATCC‐CRL‐11147, ATCC, Manassas, VA, USA), HTB63 (ATCC, Cat# ATCC‐HTB‐63), and WM852 (Cat# WC00065, Coriell Cell Repositories, Camden, NJ, USA) human melanoma cells were cultured in DMEM, McCoy’s 5a medium, and RPMI medium (HyClone, GE Healthcare, Marlborough, MA, USA), respectively, supplemented with 10% fetal bovine serum (FBS) (Sigma, Hamburg, Germany), 5 U·mL^−1^ penicillin, 0.5 U·mL^−1^ streptomycin, and 2 mm glutamine. All cell lines were regularly tested for mycoplasma contamination (EZ‐PCR kit by Biological Industries, Bet Haemek, Israel). The cells were cultured in a humidified incubator at 37 °C with 5% CO_2_.

### 2.2 Western blotting

Protein extraction and concentration determination were performed as previously described [7]. Briefly, equal amounts of total protein (30–45 μg) were prepared in 4× Laemmli buffer and heated to 95 °C for 5 min prior to being loaded on SDS‐PAGE gels. After the proteins were separated and transferred to PVDF membranes, the membranes were blocked and subsequently probed with the following antibodies: anti‐phospho‐ERK1/2 (1 : 1000) from Cell Signaling (Danvers, MA, USA), anti‐WNT5A (1 : 100) and anti‐RND3 (1 : 1000) from R&D Systems (Minneapolis, MN, USA), and anti‐MLC (phospho Ser 20) (1 : 1000), anti‐CD146 (1 : 1000), and anti‐β‐actin (1 : 30 000) from Abcam (Cambridge, UK). After washing, the membranes were incubated with either HRP‐conjugated rabbit anti‐goat secondary antibodies or HRP‐conjugated goat anti‐rabbit/mouse secondary antibodies (Dako, Glostrup, Denmark). Following a second wash, the separated protein bands were visualized using Immobilon™ Western Chemiluminescence HRP substrate (Millipore, Burlington, MA, USA) and imaged and analyzed using the ChemiDoc™ imaging system (Bio‐Rad Laboratories Inc., San Francisco, CA, USA). Densitometric quantification of relative protein expression was carried out by calculating the intensity of each band using image lab 6.0 software (Bio‐Rad Laboratories Inc.).

### 2.3 siRNA transfection and treatment

Transient siRNA transfections were performed with Lipofectamine 2000 reagent (Invitrogen, Carlsbad, CA, USA) according to the manufacturer's instructions. The following siRNA oligonucleotides were used at the indicated concentrations: anti‐WNT5A siRNA #1 (s14871; 50 nm), anti‐WNT5A siRNA #2 (s14872; 50 nm), and negative control siRNA #1 (#4390843; 50 nm) (Invitrogen). Anti‐RND3 SMARTPool siRNA (L‐007794‐00‐0050; 100 nm) was acquired from Horizon Discovery (Cambridge, UK). After 12 h, the transfection medium was replaced with fresh cell culture medium supplemented with 10% FBS, and the cells were subsequently allowed to grow for 24 or 48 h prior to analysis or further treatment. For BRAF inhibitor treatment, following siRNA treatment for 24 h, HTB63 cells were treated with 1 µm PLX4720 (Vemurafenib) (Selleckchem, Munich, Germany, Cat# S1267) or DMSO for the final 24 h of siRNA transfection. For C59 (Abcam, Cat#: ab142216) treatment, HTB63 and WM852 cells were incubated with 1 μm C59 or vehicle (DMSO) control in either collagen I gels for spheroid invasion (see Spheroid invasion assay) or while growing subconfluently in a 6‐well plate for the RhoA activity assay and western blotting (see Western blotting).

### 2.4 Spheroid invasion assay

3D spheroids were generated from subconfluent melanoma cells as previously described [6]. Briefly, a cell suspension mixture composed of cells and methylcellulose was prepared (20% methylcellulose, Cat. M6385 (Sigma)). Droplets (20 µL) containing 2000 HTB63 cells or 5000 WM852 cells were transferred onto the inner surface of a cell culture dish, which was then turned upside‐down. PBS was added to the bottom of the culture dish to prevent evaporation. After 24 h, both cell lines had formed spheroids. These spheroids were then embedded in a 2 mg·mL^−1^ rat‐tail collagen type I (Gibco, Carlsbad, CA, USA) solution, which contained DMEM (A2058 cells), McCoy’s 5A medium (HTB63 cells), or RPMI medium (WM852 cells), 5% NaHCO_3,_ and 10% FBS. One spheroid was embedded per well of a 48‐well plate, and once the collagen had polymerized, the appropriate medium was added. WNT5A‐dependent migration and morphology in collagen culture were tested in the presence of recombinant WNT5A (0.4 µg·mL^−1^; R&D Systems) or 0.1% BSA alone, which was added directly after spheroid embedding. RhoA‐dependent migration in collagen culture was tested in the presence of Rhosin (10 µm; Calbiochem, San Diego, CA, USA), which was added directly after spheroid embedding. Cdc42/Rac1‐dependent invasion was tested using Cdc42 and Rac1 activator II (0.5 units·mL^−1^; Cytoskeleton, Inc.), which was added directly after spheroid embedding. WNT5A‐dependent invasion was also inhibited using 200 μm of the WNT5A antagonistic peptide Box5 (Calbiochem [7]). Invasion type and efficacy were measured by endpoint analysis after 48 h. Images were taken using an Olympus IX81 microscope with a UPLFLN 4×/0.13 PHL objective and analyzed with cellsens software (Olympus, Tokyo, Japan).

### 2.5 3D Morphology assay

Melanoma cells were trypsinized, washed in complete medium, and counted, and 10^5^ cells were mixed with 500 μL of 2 mg·mL^−1^ collagen I (Gibco) in complete medium. A total of 250 µL of the collagen‐cell suspension was added to each well of a 24‐well plate, allowed to polymerize at 37 °C for 30 min, and subsequently overlaid with the appropriate complete medium. After 24 h, the 3D morphology of cells in collagen was analyzed using an Olympus IX81 microscope (UPLFLN 20×/0.50 objective). Cell morphology was classified on the basis of the elongation index. The elongation index was calculated as the length divided by the width. Cells with an elongation index more than 2 were considered mesenchymal cells, and cells with an index of 1–2 were considered amoeboid cells [3]. Dividing cells were excluded from the analysis. At least 250 cells were analyzed for each experiment.

### 2.6 Collagen adhesion assay

Forty‐eight‐well cell culture plates were coated with type I collagen (40 μg·mL^−1^; 37 °C; 2 h), and the wells were washed three times with DMEM. Melanoma cells were trypsinized and counted, and 5 × 10^4^ cells in suspension were added to each well and allowed to adhere for 30 min. Nonadherent cells were removed, and adherent cells were fixed in 70% cold ethanol. The cells were then stained in a 1% crystal violet/10% methanol solution at room temperature for 15 min. The cells were then imaged using an Olympus IX81 microscope (UPLFLN 20×/0.50 objective) and counted.

### 2.7 Rho GTPase activation assay

RhoA, Rac1, and Cdc42 activities were measured using the RhoA/Rac1/Cdc42 GLISA Activation Assay Bundle Kit (Cytoskeleton Inc.) according to the manufacturer’s protocol.

### 2.8 Color code of the bar graph figures

HTB63 control cells are light green throughout the manuscript, and WM852 control cells are light blue. siWNT5A 1 and siWNT5A 2 are dark blue and blood red, respectively, throughout the manuscript. Figure 4, diagonally striped bars are cells treated with the Cdc42/Rac1 activator II. The horizontally striped bars in Figs 5 and 6 are cells treated with the BRAF inhibitor PLX4720. In addition, in Fig. 6 the orange bars represent cells treated with C59; black bars represent cells treated with Rhosin; bright red bars represent cells treated with Box5; and yellow bars represent cells treated with Box5 and Rhosin.

### 2.9 Statistical analysis

Except stated otherwise, all experiments were independently repeated 4–6 times and the results are presented as means ± S.D. The data were analyzed with graphpad prism 8 software (San Diego, CA, USA) using two‐tailed Student’s *t*‐test for comparing 2 groups or ANOVA followed by the appropriate post hoc statistical test for comparing multiple groups, as indicated in the figure legends. Differences with *P* < 0.05 were considered statistically significant.

## 3 Results

**Fig. 1:**
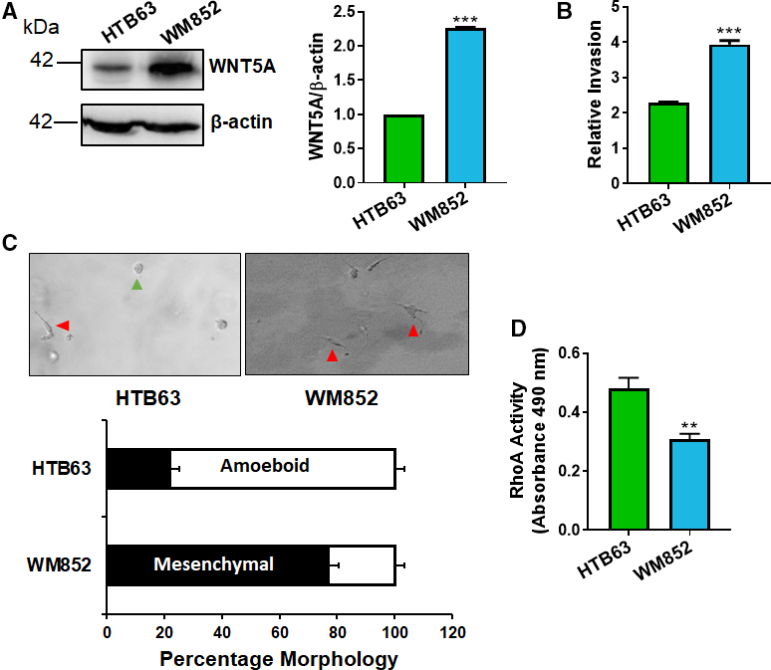
WNT5A expression is related to melanoma cell invasion, morphological phenotype, and RhoA activity. (A) Western blot analysis showing endogenous WNT5A protein expression in the human melanoma cell lines HTB63 and WM852. β‐Actin was used as a loading control. Densitometric analyses of WNT5A and β‐actin expressions were performed, and the data were then normalized to the level of β‐actin. A representative blot from 4 independent experiments is shown. (B) Human HTB63 and WM852 melanoma cells were grown as spheroids and embedded in 2 mg·mL^−1^ of collagen I matrix; invasion was analyzed after 48 h. Representative images of 6 independent experiments are shown for each cell line. (C) The 3D morphology of human HTB63 and WM852 cells was analyzed by mixing a single cell suspension of melanoma cells with 2 mg·mL^−1^ of collagen I matrix for 24 h. Representative images from 4 independent experiments are shown for each cell line; mesenchymal ■ (red arrows); amoeboid □ (green arrows). (D) A RhoA activity assay kit was used to analyze the levels of active RhoA in human melanoma HTB63 and WM852 cells. The results from the accumulated data are presented as the mean ± SD (*n* = 4 for A, C; *n* = 6 for B, D); significance was calculated using an unpaired two‐tailed Student’s *t*‐test; **P < 0.01, ***P < 0.001.

We analyzed WNT5A levels in two melanoma cell lines, that is, HTB63 and WM852 cells. A high level of WNT5A was observed in WM852 cells, and a low level was observed in HTB63 cells (Fig. 1A). As WM852 cells had a higher level of WNT5A than HTB63 cells, they exhibited a greater invasion than HTB63 cells (Fig. 1B). To validate this observation, we then analyzed the difference in invasion in the presence of recombinant WNT5A (rWNT5A) and found that WNT5A increased the spheroid invasion of HTB63 cells, but not of WM852 cells, which already produce copious levels of WNT5A, in a three‐dimensional (3D) collagen matrix (Fig. S1A). Next, we analyzed the morphology of the cells in the 3D collagen I matrix and found that HTB63 cells with limited WNT5A expression had a predominantly amoeboid morphology, whereas WM852 cells with high WNT5A expression exhibited a predominantly mesenchymal morphology (Fig. 1C). To confirm these findings, we tested the effect of rWNT5A on HTB63 cell morphology and found that it significantly increased the percentage of mesenchymal HTB63 cells (Fig. S1B). As expected, our results also showed that compared to WM852 cells, HTB63 cells exhibited higher activity of the small GTPase RhoA, which is commonly associated with the amoeboid mode of migration [23] (Fig. 1D).

**Fig. 2:**
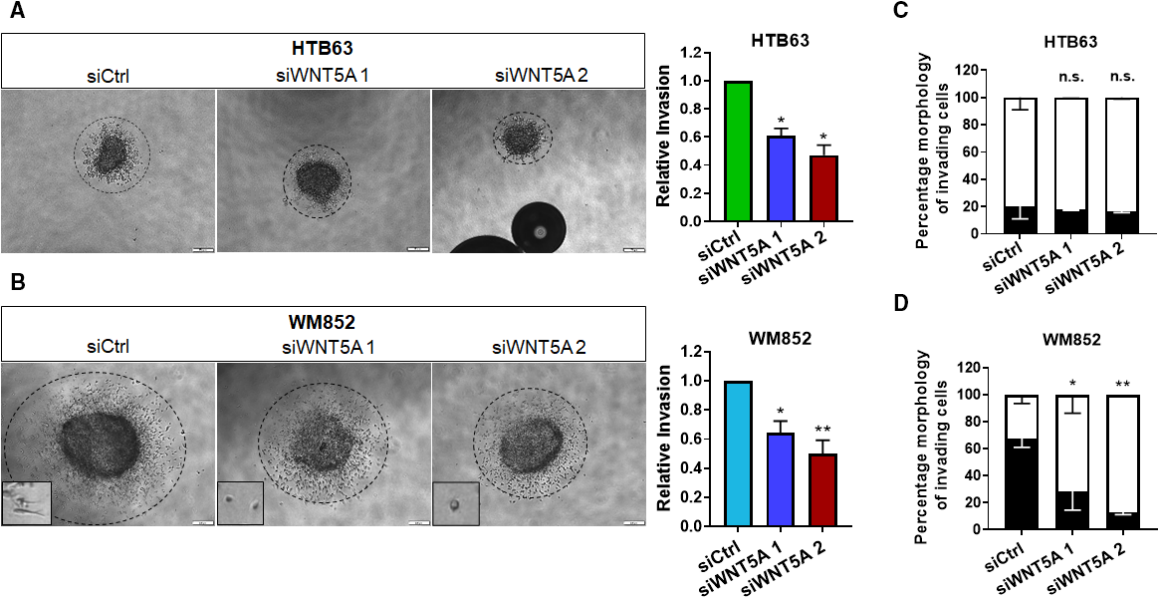
WNT5A downregulation inhibits melanoma cell invasion and promotes amoeboid morphology. (A, B) The spheroid invasion assay showing the invasion of human HTB63 (a) and WM852 (B) cells transfected with either control siRNA (siCtrl; 50 nm) or anti‐WNT5A siRNA (siWNT5A 1 or siWNT5A 2; 50 nm) that were grown as spheroids and embedded in 2 mg·mL^−1^ collagen I matrix. The panels and insets show representative images of invading cells, as well as cell at the edge of the spheroids, from 6 independent experiments. (C, D) The morphology (mesenchymal ■; amoeboid □) of individually invading HTB63 (C) and WM852 (D) melanoma cells was analyzed from the embedded spheroids in (A) and (B); the scale bars indicate 200 µm. All results (A–D) from the accumulated data are presented as the mean (*n* = 6) ± SD; significance was calculated using one‐way ANOVA with Dunnett’s *post* *hoc* test; n.s., not significant, *P < 0.05, **P < 0.01.

As the addition of rWNT5A had no effect on the invasion of WM852, we analyzed the dependence of invasion on WNT5A via a knockdown approach. Using two different siRNAs targeting WNT5A, we successfully knocked down WNT5A in HTB63 and WM852 cells (Fig. S2). We then analyzed invasion using the 3D spheroid invasion method and observed that knockdown of WNT5A caused a significant decrease in the invasion of both melanoma cell lines (Fig. 2A,B). Interestingly, when we analyzed individually invading cells from the spheroids, we observed no change in morphology following WNT5A knockdown, as the HTB63 cells were already amoeboid (Fig. 2C). In contrast, we observed that WNT5A downregulation led to a more amoeboid mode of migration in the remaining and still invading WM852 cells via a shift from a predominantly mesenchymal mode of migration (Fig. 2D).

**Fig. 3:**
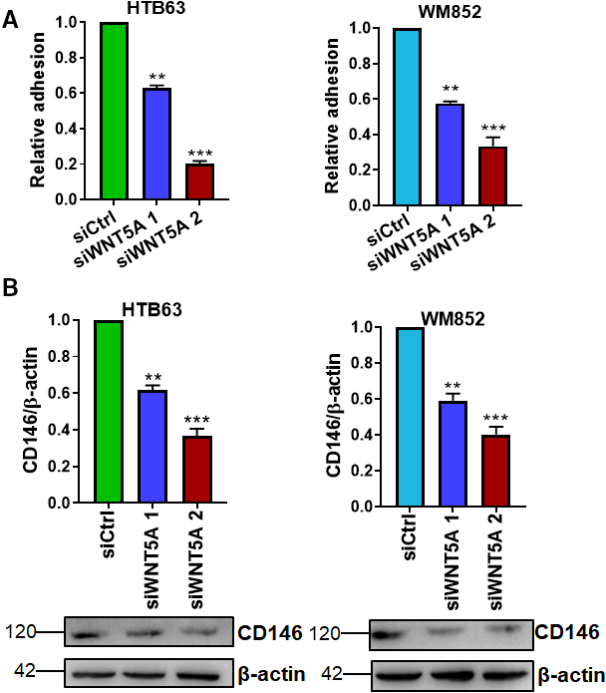
WNT5A downregulation inhibits melanoma cell adhesiveness. (A) Human HTB63 and WM852 melanoma cells transfected with either control siRNA (siCtrl; 50 nm) or anti‐WNT5A siRNA (siWNT5A or siWNT5A 2; 50 nm) were tested and analyzed for their adherence ability to 40 µg·mL^−1^ collagen I‐coated plates. Adherent cells were fixed and stained with crystal violet, then counted. (B) Western blot analyses were performed to determine CD146 protein expression levels in HTB63 and WM852 cells following transfection with either negative control siRNA (siCtrl; 50 nm) or anti‐WNT5A siRNA (siWNT5A 1 or siWNT5A 2; 50 nm). β‐Actin was used as a loading control. Densitometric analysis of CD146 and β‐actin expressions was performed, and the data were then normalized to the level of β‐actin. From 6 independent experiments, we show a representative image of CD146 expressions and β‐actin control expressions for each cell line. All results from the accumulated data (A–B) are presented as the mean (*n* = 6) ± SD; significance was calculated using one‐way ANOVA with Dunnett’s *post* *hoc* test; **P < 0.01, ***P < 0.001.

It is well described that amoeboid cells have a lower adhesive property than mesenchymal cells [24, 25]. Consequently, we further validated these results by analyzing the adhesive properties of the cells. HTB63 melanoma cells had lower adhesive capacity (39 ± 1%) than WM852 cells. However, upon WNT5A knockdown, both cell lines showed reduced adhesion to a collagen matrix (Fig. 3A). These findings are in agreement with the observation that WNT5A knockdown led to reduced invasion of both cell lines and may have been related to the increase in the number of amoeboid WM852 cells since amoeboid cells are generally considered to be less adherent than mesenchymal cells [23]. Recently, it was shown that CD146/MCAM (melanoma cell adhesion molecule) is essential for melanoma cell adhesion and metastasis [26]. Here, we observed that WNT5A knockdown was associated with reduced CD146/MCAM levels (Fig. 3B). CD146/MCAM expression increases gradually with melanoma progression and has been shown to be higher in metastatic melanoma cells than in nonmetastatic melanoma cells [26].

**Fig. 4:**
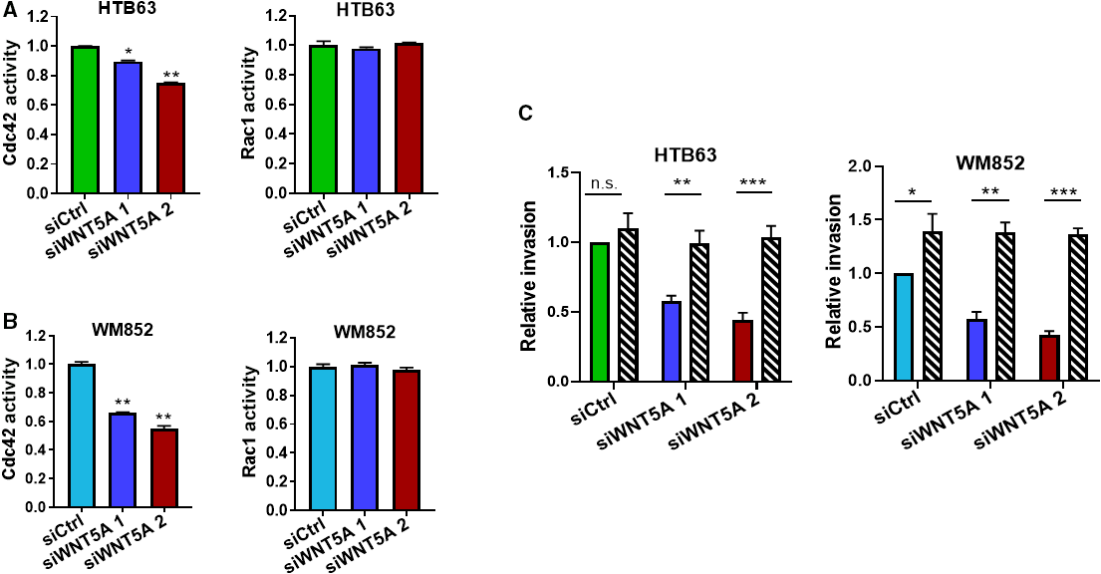
WNT5A knockdown inhibits Cdc42, but not Rac1, activation. (A, B) Cdc42 and Rac1 activities were measured in HTB63 (A) and WM852 (B) melanoma cells transfected with either control siRNA (siCtrl; 50 nm) or anti‐WNT5A siRNA (siWNT5A 1 or siWNT5A 2; 50 nm). Cdc42 and Rac1 activities were measured using the RhoA/Cdc42/Rac1 GLISA kit. Activity levels were normalized to those in siCtrl‐transfected cells. (C) HTB63 and WM852 melanoma cells transfected with either control siRNA (siCtrl; 50 nm) or anti‐WNT5A siRNA (siWNT5A 1 or siWNT5A 2; 50 nm) were grown as spheroids and embedded in 2 mg·mL^−1^ collagen I matrix, in the absence (colored) or presence (striped) of Cdc42/Rac1 activator II. All results (A–C) from the accumulated data are presented as the mean (*n* = 6) ± SD, significance was calculated using one‐way (A, B) and two‐way ANOVA (C) with Dunnett’s and Tukey’s *post hoc* tests, respectively; n.s. not significant, *P < 0.05, **P < 0.01, ***P < 0.001.

The fact that different Rho GTPases have been closely implicated in the regulation of cell morphology and invasion [24, 27], we next investigated the observed effects described above, by performing Rho GTPase activity assays for Cdc42, Rac1, and RhoA. Upon siRNA knockdown of WNT5A, we found significantly decreased Cdc42 activity in both cell lines; however, there were no observed significant effects on Rac1 activity in either cell line (Fig. 4A,B). We then explored whether the reduced activity of Cdc42 is responsible for the impaired invasion of melanoma cells upon reduced WNT5A expression through gain‐of‐function experiments with the Cdc42 and Rac1 activator II. We found that melanoma cell invasion, which had been inhibited by WNT5A knockdown in both cell lines, was restored in these cells by the addition of this activator (Fig. 4C, striped columns).

**Fig. 5:**
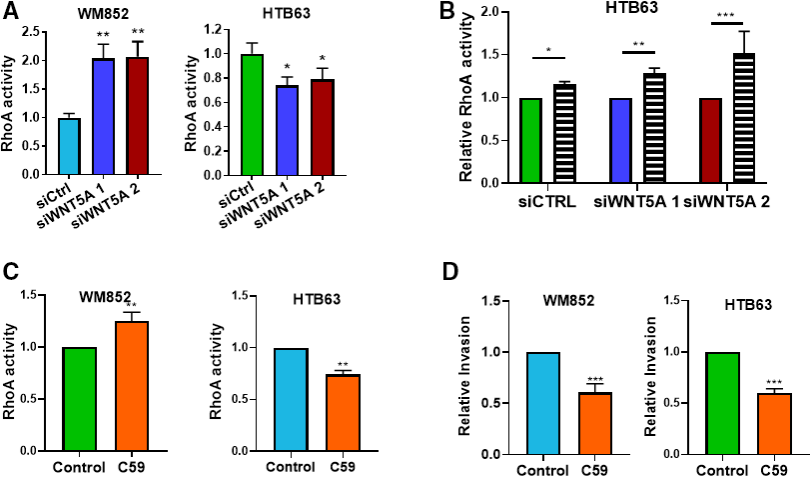
WNT5A knockdown increases RhoA activation in WM852 cells but not in HTB63 cells unless they are treated with a BRAF inhibitor. (A) RhoA activity was measured in HTB63 and WM852 melanoma cells transfected with either control siRNA (siCtrl; 50 nm) or anti‐WNT5A siRNA (siWNT5A 1 or siWNT5A 2; 50 nm) using a RhoA activity assay GLISA kit. Activity levels were normalized to those in siCtrl‐transfected cells. (B) The RhoA activity assay kit was also used to determine RhoA activity in HTB63 melanoma cells transfected with either control siRNA (siCtrl; 50 nm) or anti‐WNT5A siRNA (siWNT5A 1 or siWNT5A 2; 50 nm) and incubated in either vehicle alone (DMSO, colored) or the BRAF inhibitor PLX4720 (striped). The data were normalized to the data for each individual control. (C) RhoA activity was measured in WM852 and HTB63 cells treated with vehicle (control) or 1 µm C59 inhibitor for 24 h. (D) The spheroid invasion assay showing the invasion of human HTB63 and WM852 cells treated with either vehicle (control) or 1 µm C59. HTB63 and WM852 cells were grown as spheroids and embedded in 2 mg·mL^−1^ collagen I matrix, in the presence or absence of the C59 inhibitor. Spheroids were grown from 48 h and invasion was visualized using microscopy and subsequently measured using imagej. All results from the accumulated data (A–D) are presented as the mean (6 = 6) ± SD; significance was calculated using one‐way (A) and two‐way ANOVA (B) with Dunnett’s and Tukey’s *post hoc* tests, respectively, or unpaired two‐tailed Student’s *t*‐test (C–D). *P < 0.05, **P < 0.01, ***P < 0.001.

With regard to RhoA activity, we made the very interesting and logical observation that WNT5A knockdown in WM852 cells caused an increase in the activity of RhoA, a GTPase closely associated with the amoeboid mode of migration (Fig. 5A), that corresponded to the observed mesenchymal‐to‐amoeboid transition in these cells (Fig. 2D). Following knockdown of WNT5A in HTB63 cells, which already exhibit an amoeboid morphology, we did not observe a mesenchymal‐to‐amoeboid transition (Fig. 2C), and consequently, no further increase in RhoA activity (Fig. 5A) was noted. In fact, WNT5A knockdown in HTB63 cells caused a clear decrease in RhoA activity (Fig. 5A). Reduced WNT5a signaling clearly had opposite effects on Rho activity levels in WM852 and HTB63 melanoma cells. One possible explanation for these opposing effects could be related to the fact that WM852 cells express wild‐type BRAF, whereas HTB63 cells express the active BRAF*^V600E^* mutation. To explore the effects of this difference, we tested RhoA activity changes in response to reduced WNT5A expression in HTB63 cells in the absence and presence of the BRAF inhibitor PLX4720. Our data clearly showed that in the presence of this BRAF inhibitor, RhoA activity changed from being decreased (Fig. 5A) to being increased (Fig. 5B) in response to reduced WNT5A signaling, the latter response being similar to that observed in WM852 cells (Fig. 5A). To confirm that the observed results following WNT5A knockdown are due to decreased WNT5A secretion and not just reduced expression, we analyzed HTB63 and WM852 RhoA activities and their spheroid invasion in the presence of the Wnt inhibitor, C59. C59 inhibits porcupine, a membrane‐bound *O*‐acyltransferase that is necessary for the secretion of Wnts and their biological activities. We found that, as with the WNT5A knockdown, C59 increased RhoA activity in WM852, but decreased it in HTB63 cells (Fig. 5C). Also, in agreement with our siWNT5A data, C59 significantly decreased WM852 and HTB63 spheroid invasion (Fig. 5D).

**Fig. 6:**
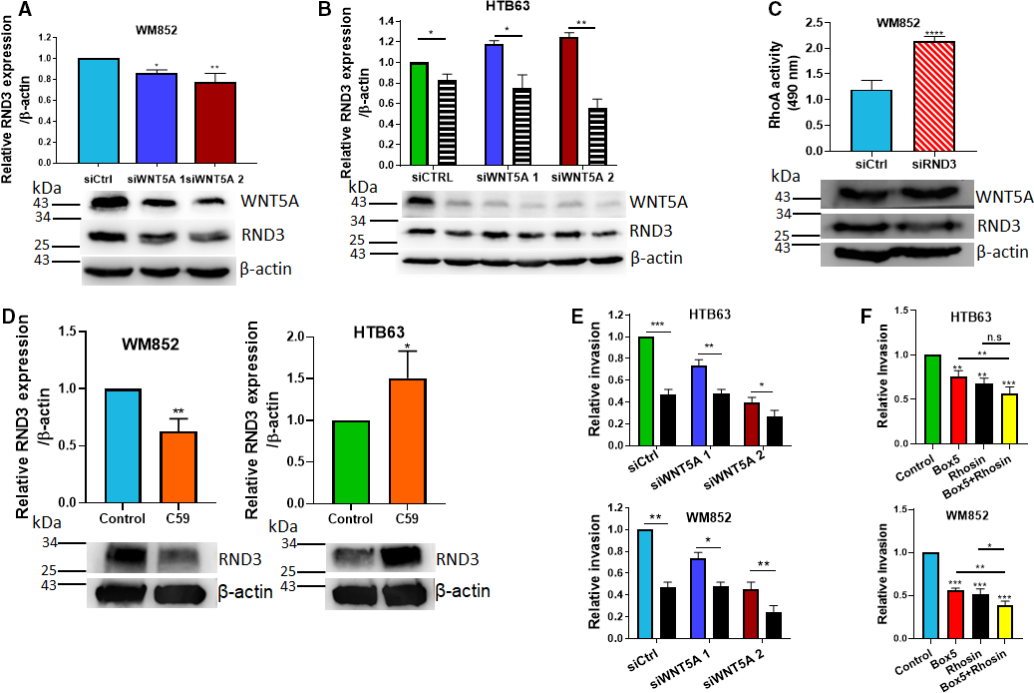
WNT5A differentially regulates RND3 expression in melanoma cells. (A, B). Western blot analyses were performed to determine the expression of RND3, WNT5A, and β‐actin in WM852 (A) and HTB63 (B) melanoma cells following transfection with either control siRNA (siCtrl; 50 nm) or anti‐WNT5A siRNA (siWNT5A 1 or siWNT5A 2; 50 nm) and for the experiments outlined in (B), in the presence of vehicle (DMSO, colored) or the BRAF inhibitor PLX4032 (striped). β‐Actin was used as a loading control. Densitometric analyses of RND3, WNT5A, and β‐actin expressions (A, B) were performed, and the data were then normalized to the level of β‐actin. Representative blots are shown from 6 independent experiments for each cell line. (C) A RhoA activity assay kit was used to determine RhoA activity, and Western blot analyses were performed to evaluate the expression of RND3, WNT5A, and β‐actin in WM852 cells transfected with either control siRNA (siCtrl; 100 nm) or anti‐RND3 SMARTpool siRNA (100 nm). The Western blot analyses of RND3 and WNT5A expression are shown as representative blots from 6 independent experiments. (D) Western blot analyses were performed to determine RND3 protein expression levels in WM852 and HTB63 cells following treatment with either vehicle (control) or 1 µm C59. β‐Actin was used as loading controls. Densitometric analysis of RND3 and β‐actin expressions were performed, and the RND3/β‐actin ratios were normalized to that of control. Representative images of RND3 expressions and their β‐actin controls are shown from 4 independent experiments for each cell line. (E) The spheroid invasion assay was used to evaluate and analyze the invasion of HTB63 and WM852 cells transfected with either control siRNA (siCtrl; 50 nm) or anti‐WNT5A siRNA (siWNT5A 1 and siWNT5A 2; 50 nm) in the absence (colored) or presence (black) of the RhoA‐GEF inhibitor Rhosin. (F) The spheroid invasion assay, where HTB63 and WM852 cells were grown as spheroids and embedded in 2 mg·mL^−1^ of collagen I, was used to evaluate and analyze the invasion of HTB63 and WM852 cells treated with 200 µm Box5, 10 µm Rhosin, or a mixture of both. Invasion was analyzed after 48 h, visualized using microscopy, and measured using imagej. All results from the accumulated data (A–F) are presented as the mean ± SD, (*n* = 4 for D and *n* = 6 for A, B, C, E, F); statistical significance was calculated using one‐way (A, F) and two‐way ANOVA (B, E) with Dunnett’s and Tukey’s *post hoc* tests, respectively, or an unpaired two‐tailed Student’s *t‐*test (C, D); n.s. not significant, *P < 0.05, **P < 0.01, ***P < 0.001.

We next addressed the strange observation that reduced levels of the WNT5A ligand resulted in increased RhoA signaling in WM852 cells and in HTB63 cells treated with the BRAF inhibitor PLX4720. Our initial investigation failed to demonstrate reduced phosphorylation and activation of a RhoA GTPase; therefore, we addressed the possible involvement of altered expression of the atypical Rho GTPase RND3, which has no GTPase activity but serves as an inhibitor of RhoA activity [28, 29]. The RND3 is a member of the Rnd family of proteins, which are a subgroup of small Rho GTPases, and comprise of three proteins in humans: RND1, RND2, and RND3. RND3 expression and function have been highlighted recently in cancers; however, there are limited studies in melanoma [30]. The RND3/RhoE GTPase, also known as an atypical GTPase due to its lack of GTPase activity, negatively regulates the RhoA/ROCK signaling pathway [29]. Interestingly, such a regulation of RhoA signaling by RND3 was shown to be present in BRAF*^V600^* cells [28, 29]. In the present study, we found that knockdown of WNT5A expression significantly decreased the expression of RND3 in wild‐type BRAF‐expressing WM852 cells (Fig. 6A), which is in good agreement with the increased RhoA signaling observed under these conditions (Fig. 5A). In contrast, reduced WNT5A signaling in BRAF‐mutant HTB63 cells resulted in an increase in the expression of RND3 (Fig. 6B). However, we also found that in the presence of the BRAF inhibitor PLX4720 the HTB63 cells the effect of WNT5A knockdown was opposite with a significant decrease in RND3 expression (Fig. 6B). These data are consistent with the ability of the BRAF inhibitor to alter the response to a WNT5A knockdown by causing an increase in RhoA activity under similar conditions (Fig. 5C). In control experiments, we show that the PLX4720 BRAF inhibitor effectively inhibited ERK1/2 phosphorylation in HTB63 cells (Fig. S3). These results show for the first time that the atypical GTPase RND3 is regulated in a WNT5A‐dependent manner (Fig. 6A,B), and our observation that RND3 expression and RhoA activity are equally dependent on BRAF activity in melanoma cells provides further evidence for the crucial involvement of RND3 in the regulation of RhoA activity in melanoma cells. This conclusion was supported by the finding that siRNA‐mediated knockdown of RND3 in WM852 cells led to a significant increase in RhoA activity (Fig. 6C) and by Klein and Higgins’ report that BRAF inhibition reduces the expression of RND3 and increases RhoA activation [28]. To further validate these findings, we also impaired Wnt secretion by incubation with the porcupine inhibitor C59 and found that in WM852 cells it decreased RND3 expression, whereas in HTB63 cells it increased RND3 expression (Fig. 5D).

Finally, we explored the therapeutic potential of the above observations, by investigating the effects of the RhoA‐GEF (guanine exchange factor) inhibitor Rhosin, which selectively inhibits RhoA activity [31], on the invasive capacity of siControl‐ and siWNT5A‐treated melanoma cells. We found that in both cell lines, dual targeting of RhoA and WNT5A resulted in a greater decrease in invasion than targeting WNT5A alone (Fig. 6E, Fig. S4). To further validate these findings, we performed similar experiments but instead of using siWNT5A treatment, we targeted WNT5A with the WNT5A inhibitor peptide Box5 [7]. The results suggest that dual targeting of WNT5A and RhoA activity is a more efficient approach to impair melanoma cell invasion than inhibiting each target alone (Fig. 6F, Fig. S5).

Taken together, our results suggest that WNT5A downregulation inhibits Cdc42‐dependent melanoma cell invasion in 3D collagen matrices. However, this impairment of WNT5A signaling appears to trigger a compensatory mechanism to maintain the invasive migration of BRAF wild‐type and BRAF^V600^ mutated melanoma cells treated with a BRAF inhibitor, through a RND3‐RhoA‐induced transition from a mesenchymal to an amoeboid mode of invasion. Therefore, dual targeting of both WNT5A and RhoA signaling is more effective in inhibiting the invasion of these melanoma cells than targeting either of these signaling molecules alone.

## 4 Discussion

The main aim of this study was to analyze the potential role of WNT5A in melanoma cell plasticity, in particular mesenchymal‐to‐amoeboid transition. The noncanonical ligand WNT5A is associated with higher tumor grade and increased metastatic capabilities of melanoma cells [32]. WNT5A is differentially expressed during various stages of melanoma, exhibiting a limited increase in expression during benign nevus and the vertical growth phase and a greater increase in expression during the metastatic phase [33]. Thus, WNT5A has been suggested as a marker of progressive melanoma and a potential therapeutic target [34]. Although there have been numerous reports suggesting that WNT5A plays an essential role in melanoma invasion, the exact mechanism whereby WNT5A regulates this progressive behavior of melanoma cells has not been fully elucidated. Here, we show that impaired WNT5A signaling not only significantly decreases melanoma cell invasion but can also promote a more rounded, amoeboid invasive phenotype. This mesenchymal‐to‐amoeboid transition is in good accordance with our observation that WNT5A knockdown does not completely inhibit melanoma cell invasion and suggests the presence of a compensatory mechanism through which melanoma cells are able to invade a 3D collagen matrix.

Cancer cells are able to transition from a proteolytic mesenchymal mode of invasion to a protease‐independent amoeboid mode of invasion that can sustain cancer cell dissemination in the presence of cancer treatments [3]. In 3D *in vitro* and *in vivo* environments, mesenchymal cells develop an elongated, spindle‐shaped morphology that is coupled to ECM remodeling and cleavage through the release of MMPs and other proteases by tumor cells [35]. The WM852 melanoma cells used in the present study exhibit mesenchymal behavior in a 3D collagen matrix, moving as individual spindle‐shaped cells. As the diameter of the elongated mesenchymal cells is generally greater than the pore size within the collagen fiber network [36], contact‐dependent focal remodeling and degradation of ECM by mesenchymal cells allow them to invade by reducing the physical resistance of the intact ECM barrier [37]. In some cancer cell types, invasion results from a more dynamic, temporary, and less‐defined cell–matrix contact, which is associated with a rounded amoeboid tumor cell shape [38]. Our present data reveal that the rounded amoeboid morphology exhibited by HTB63 cells is associated with a low level of WNT5A expression and high RhoA activity in these cells. Cells demonstrating a rounded amoeboid morphology generally maintain their motion, which includes rapid, low‐affinity gliding that is accompanied by shape change, allowing them to squeeze through the pores in the matrix [39]. Therefore, invasion of amoeboid cells also appears to be independent of matrix remodeling/cleavage and would therefore be insensitive to protease inhibitor treatment [3].

There is a striking difference in migration mode between HTB63 and WM852 melanoma cells that correlates with their different levels of WNT5A expression. Knockdown of WNT5A expression not only decreased melanoma cell invasion but also resulted in a significant reduction in Cdc42 activity but no change in Rac1 activity in either cell line. To analyze whether impaired Cdc42 signaling is responsible for the reduced invasion caused by WNT5A knockdown in these melanoma cells, we stimulated the cells with a Cdc42 and Rac1 activator. The fact that this activator restores the invasive property of WNT5A knockdown melanoma cells led us to conclude that Cdc42 signaling is essential for the WNT5A‐driven invasion of melanoma cells regardless of whether they invade via an amoeboid or mesenchymal mode of migration. This finding is consistent with previous reports that Cdc42 activation and signaling play important roles in melanoma cell invasion [40].

Surprisingly, WNT5A knockdown induced an unexpected increase in RhoA activity in the predominantly mesenchymal WM852 cell line. As expected for this signaling event, there was a parallel mesenchymal‐to‐amoeboid transition in these cells. The notion that an amoeboid phenotype increases tumor cell invasion [41] led us to believe that the mesenchymal‐to‐amoeboid transition of WM852 cells constitutes a compensatory mechanism in response to the reduced Cdc42‐dependent invasion of these cells. In the already amoeboid HTB63 cells, on the other hand, there was no phenotypic switch, suggesting that impaired WNT5A signaling specifically induces rounded amoeboid migration in melanoma cells that exhibit a predominantly elongated mesenchymal mode of migration. However, rWNT5A stimulation of HTB63 cells, which have a low endogenous expression of WNT5A, triggered a distinct amoeboid‐to‐mesenchymal transition further supporting an essential role of WNT5A signaling in the regulation of melanoma cell plasticity.

Melanoma cells with differing genotypes can exhibit different responses to inhibitors and gene targeting approaches [42]. HTB63 and WM852 cells are not only phenotypically different, as shown in Fig. 1C, but also genetically different. HTB63 cells possess an active *BRAF^V600E^* mutation, the most commonly observed mutation in melanoma patients, whereas WM852 cells do not harbor this mutation. Patients frequently treated with BRAF inhibitors, such as dabrafenib and vemurafenib, can develop resistance to these inhibitors, leading to metastatic spread and worse outcomes [43]. Here, we also show the importance of Rho‐family GTPases in the regulation of melanoma cell invasion. Although Rho GTPases are not frequently mutated, their activity and expression are usually altered in tumor progression [29]. The RND3 GTPase, termed the atypical GTPase due to its lack of GTPase activity, is a negative regulator of the RhoA/ROCK signaling pathway [29]. Interestingly, a switch in RND3‐RhoA signaling was shown to be important in BRAF*^V600^* cells; however, we show here that this switch also occurs in wild‐type BRAF cells (WM852). Importantly, BRAF inhibition in HTB63 cells mirrored the effects of WNT5A downregulation on RhoA activity observed in WM852 cells. The differences between the cell lines explain the variation in the overall response to WNT5A knockdown, particularly with respect to RhoA. These mechanisms may be important for allowing cells to persist and maintain invasiveness during WNT5A inhibition. Thus, RND3‐RhoA signaling presents a possible compensatory mechanism in wild‐type BRAF and BRAF*^V600^*‐mutant melanoma cells treated with a BRAF inhibitor, suggesting that it could be a potential drug target in combination with WNT5A interference.

Furthermore, recent reports have highlighted the importance of dual targeting of mesenchymal and amoeboid cells in inhibiting melanoma cell invasiveness [44]. This is applicable to the present study, in which we found that inhibition of RhoA signaling by the RhoA‐GEF inhibitor Rhosin in WNT5A‐deficient melanoma cells resulted in an even more significant reduction in melanoma cell invasion than WNT5A knockdown alone. This supports our view that under the circumstances described above, the RhoA‐mediated mesenchymal‐to‐amoeboid transition serves to counteract the impaired invasion of WNT5A‐deficient melanoma cells.

## 5 Conclusion

In conclusion, while WNT5A‐deficient melanoma cells exhibit reduced Cdc42 signaling and invasion, we also identified a counteracting response to this reduction in invasion in BRAF wild‐type and BRAF^V600^ mutated melanoma cells treated with a BRAF inhibitor. The mechanism underlying our novel observation that WNT5A knockdown in these melanoma cells triggers a counteracting feedback response is a switch in RND3‐RhoA signaling. This feedback mechanism leads to an amoeboid phenotype that allows cells to invade. When RhoA and WNT5A signaling are simultaneously blocked in these cells, their invasion is more effectively inhibited. These results highlight the need to further develop a safe and reliable WNT5A inhibitor to be used in combination with a Rho GTPase inhibitor as a clinical treatment strategy to effectively impair melanoma cell invasion and metastasis.

## Conflict of interest

TA is a shareholder, scientific advisor, and member of the Board of WntResearch AB. This does not alter the author’s adherence to all guidelines for publication in Molecular Oncology. NJ and LÅ have no conflicts of interest to declare.

## Author contributions

NJ and TA conceived the idea of the study. NJ performed the absolute majority of the experiments in the study and wrote the first draft of the manuscript. LÅ contributed by performing complementary and explorative experiment in the study. NJ and TA discussed the results and interpreted the data. TA and NJ critically reviewed and corrected the manuscript. All authors reviewed and agreed to the information provided in the manuscript.

## Supporting information


**Fig. S1.** (A) Spheroid invasion assay showing the invasion of human HTB63 and WM852 cells grown as spheroids and then embedded into 2 mg/ml of collagen I in the absence (vehicle control, colored) or presence (0.4 µg/ml of recombinant WNT5A (rWNT5A), grey) for 48 h. Spheroid area was analyzed before and after invasion using ImageJ, and the data were then normalized to those of control HTB63 cells. Representative images are shown form 6 independent experiments for both cell lines. (B) Morphology assay of HTB63 cells embedded in 2 mg/ml of Collagen I, treated with vehicle control or 0.4 µg/ml of rWNT5A, amoeboid cells are white and mesenchymal cells are black. The results from accumulated data are presented as the mean (n = 6) ± S.D.; *P < 0.05, **P < 0.01.
**Fig. S2.** Western blot analyses showing WNT5A protein expression in HTB63 and WM852 cells transfected with either control siRNA (siCtrl; 50 nM) or anti‐WNT5A siRNA (siWNT5A 1 and siWNT5A 2; 50 nM) and incubated for 48 h. GAPDH was used as a loading control.
**Fig. S3.** Representative images of the spheroid invasion assay showing the invasion of HTB63 and WM852 cells transfected with either control siRNA (siCtrl; 50 nM) or anti‐WNT5A siRNA (siWNT5A 1 and siWNT5A 2; 50 nM) that were grown as spheroids and embedded in 2 mg/ml Collagen I, in the absence (vehicle control) or presence of the RhoA‐GEF inhibitor Rhosin for 48 h.
**Fig. S4.** Western blot analyses were performed to determine the level of phospho‐ERK1/2 in HTB63 melanoma cells following transfection with either control siRNA (siCtrl; 50 nM) or anti‐WNT5A siRNA (siWNT5A 1 or siWNT5A 2; 50 nM) and in the presence of vehicle (colored bars) or the BRAF inhibitor PLX4720 (grey bars).
**Fig. S5.** The spheroid invasion assay was used to evaluate and analyze the invasion of HTB63 and WM852 cells that were grown as spheroids and embedded in 2 mg/ml Collagen I, and treated with 10 µM Rhosin, 200 µM Box5 or a mixture of both.

## Data Availability

All data generated or analyzed during this study are included in this published article and its Supporting information file.
