# Supplementary material for: Reduced WNT5A signaling in melanoma cells favors an amoeboid mode of invasion
Source: Mol Oncol. 2021 May 15;15(7):1835–48. doi: 10.1002/1878-0261.12974 (PMC8253101; doi:10.1002/1878-0261.12974)
Supplement: Supplementary file 1 — Fig. S1. (A) Spheroid invasion assay showing the invasion of human HTB63 and WM852 cells grown as spheroids and then embedded into 2 mg/ml of collagen I in the absence (vehicle control, colored) or presence (0.4 µg/ml of recombinant WNT5A (rWNT5A), grey) for 48 h. Spheroid area was analyzed before and after invasion using ImageJ, and the data were then normalized to those of control HTB63 cells. Representative images are shown form 6 independent experiments for both cell lines. (B) Morphology assay of HTB63 cells embedded in 2 mg/ml of Collagen I, treated with vehicle control or 0.4 µg/ml of rWNT5A, amoeboid cells are white and mesenchymal cells are black. The results from accumulated data are presented as the mean (n = 6) ± S.D.; *P < 0.05, **P < 0.01. Fig. S2. Western blot analyses showing WNT5A protein expression in HTB63 and WM852 cells transfected with either control siRNA (siCtrl; 50 nM) or anti‐WNT5A siRNA (siWNT5A 1 and siWNT5A 2; 50 nM) and incubated for 48 h. GAPDH was used as a loading control. Fig. S3. Representative images of the spheroid invasion assay showing the invasion of HTB63 and WM852 cells transfected with either control siRNA (siCtrl; 50 nM) or anti‐WNT5A siRNA (siWNT5A 1 and siWNT5A 2; 50 nM) that were grown as spheroids and embedded in 2 mg/ml Collagen I, in the absence (vehicle control) or presence of the RhoA‐GEF inhibitor Rhosin for 48 h. Fig. S4. Western blot analyses were performed to determine the level of phospho‐ERK1/2 in HTB63 melanoma cells following transfection with either control siRNA (siCtrl; 50 nM) or anti‐WNT5A siRNA (siWNT5A 1 or siWNT5A 2; 50 nM) and in the presence of vehicle (colored bars) or the BRAF inhibitor PLX4720 (grey bars). Fig. S5. The spheroid invasion assay was used to evaluate and analyze the invasion of HTB63 and WM852 cells that were grown as spheroids and embedded in 2 mg/ml Collagen I, and treated with 10 µM Rhosin, 200 µM Box5 or a mixture of both. [file MOL2-15-1835-s001.docx]

**Supplemental Figures**

**A**

**
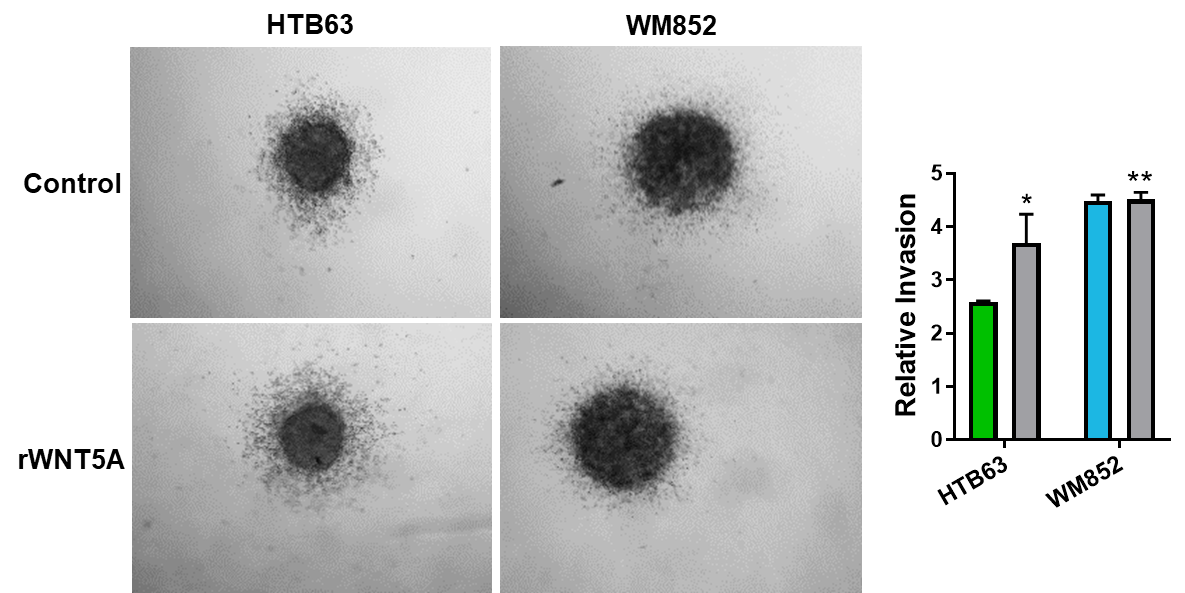
**

**
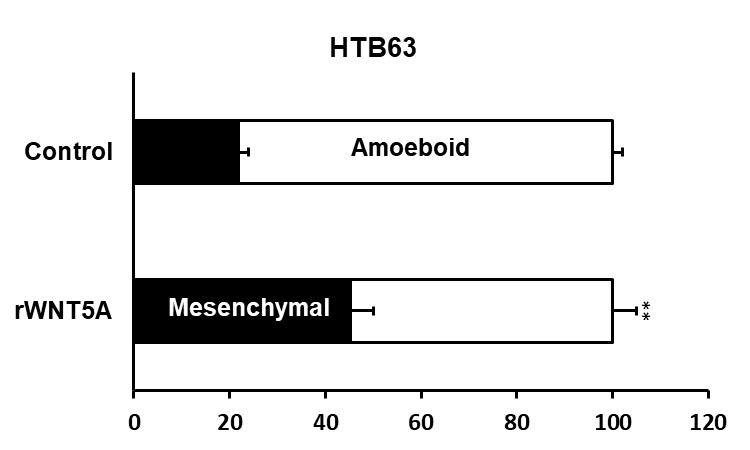
B**

**Percentage Morphology**

**Supplementary Figure 1. (A)** Spheroid invasion assay showing the invasion of human HTB63 and WM852 cells grown as spheroids and then embedded into 2 mg/ml of collagen I in the absence (vehicle control, colored) or presence (0.4 µg/ml of recombinant WNT5A (rWNT5A), grey) for 48 h. Spheroid area was analyzed before and after invasion using ImageJ, and the data were then normalized to those of control HTB63 cells. Representative images are shown form 6 independent experiments for both cell lines. (**B**) Morphology assay of HTB63 cells embedded in 2 mg/ml of Collagen I, treated with vehicle control or 0.4 µg/ml of rWNT5A, amoeboid cells are white and mesenchymal cells are black. The results from accumulated data are presented as the mean (n=6) ± S.D. ; * *P* < 0.05, ** *P* < 0.01.


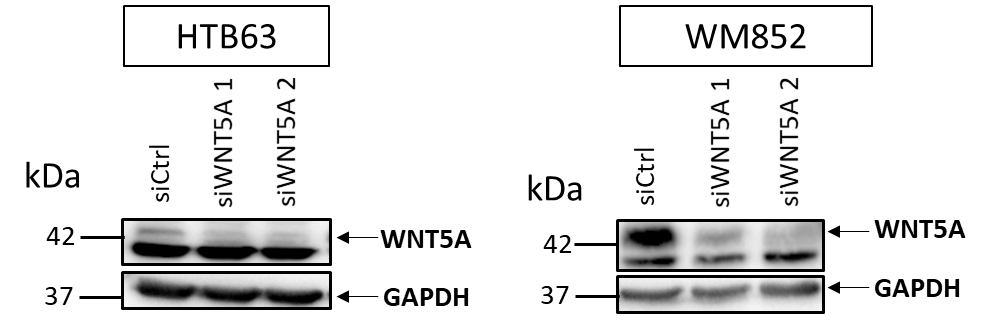


**Supplementary Figure 2.** Western blot analyses showing WNT5A protein expression in HTB63 and WM852 cells transfected with either control siRNA (siCtrl; 50 nM) or anti-WNT5A siRNA (siWNT5A 1 and siWNT5A 2; 50 nM) and incubated for 48 h. GAPDH was used as a loading control. Representative blots are shown from 4 independent experiments for both cell lines.

**
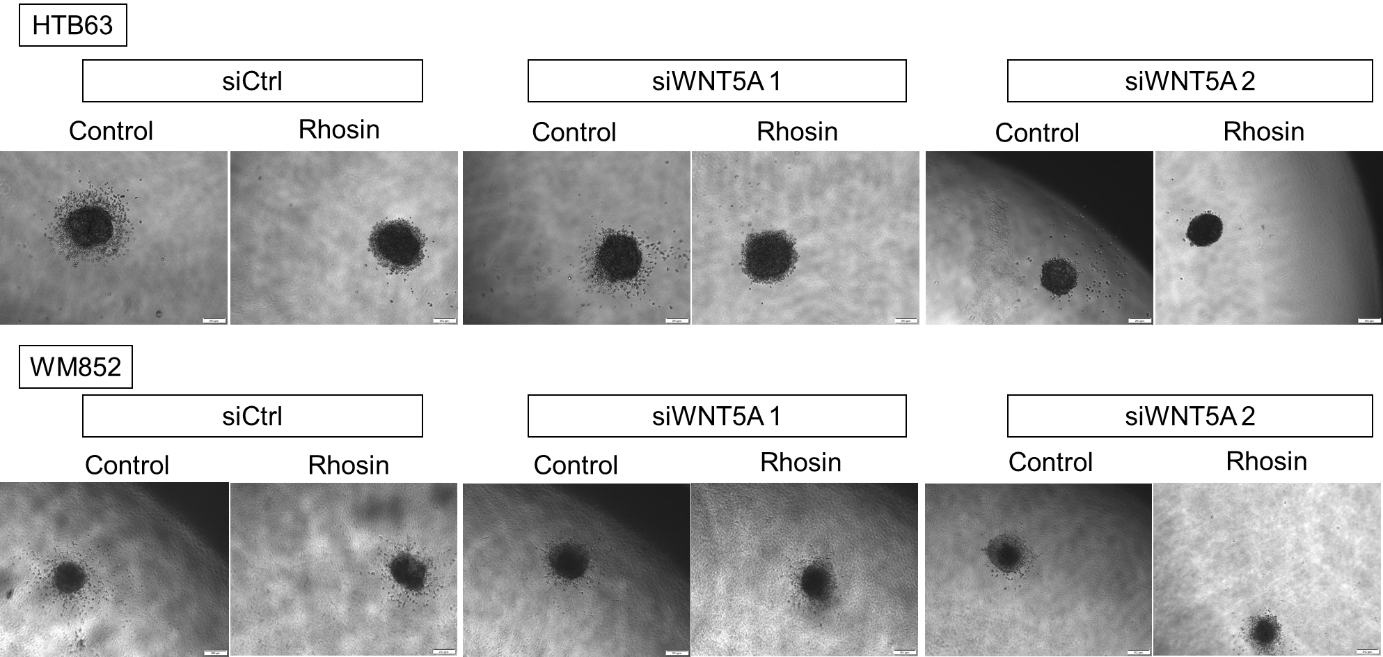
**

**Supplementary Figure 3**. Representative images of the spheroid invasion assay showing the invasion of HTB63 and WM852 cells transfected with either control siRNA (siCtrl; 50 nM) or anti-WNT5A siRNA (siWNT5A 1 and siWNT5A 2; 50 nM) that were grown as spheroids and embedded in 2 mg/ml Collagen I, in the absence (vehicle control) or presence of the RhoA-GEF inhibitor Rhosin for 48 h. Invasion was then visualized using microscopy, and representative images are shown from 6 independent experiments for both cell lines. The scale bars represent 200 µm.


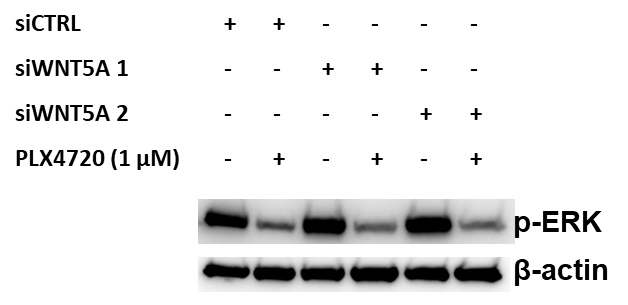

**Supplementary Figure 4**. Western blot analyses were performed to determine the level of phospho-ERK1/2 in HTB63 melanoma cells following transfection with either control siRNA (siCtrl; 50 nM) or anti-WNT5A siRNA (siWNT5A 1 or siWNT5A 2; 50 nM) and in the presence of vehicle **(**colored bars) or the BRAF inhibitor PLX4720 (grey bars). β-actin was used as a loading control. Representative blots from 4 independent experiments are shown. Densitometric analysis of phospho-ERK 1/2 levels and β-actin were performed. The results from the accumulated data are presented as the mean (4=4) ± S.D. Statistical significance was calculated using two-way ANOVA with Dunnett’s post hoc tests *** *P* < 0.0001.


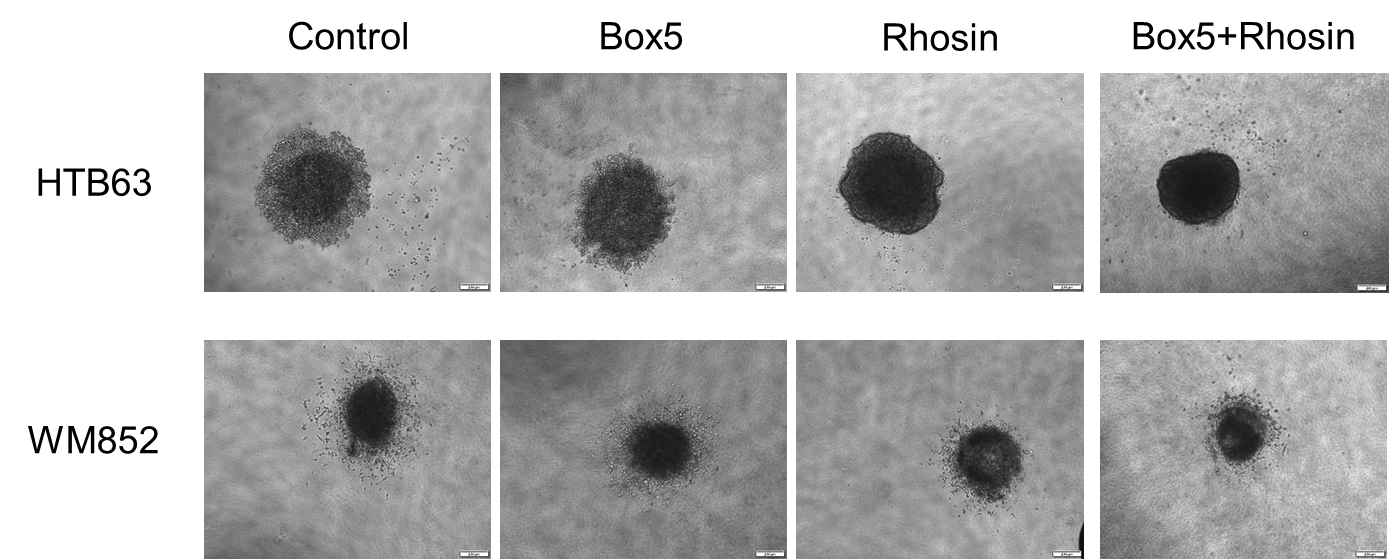


**Supplementary Figure 5.** The spheroid invasion assay was used to evaluate and analyze the invasion of HTB63 and WM852 cells that were grown as spheroids and embedded in 2 mg/ml Collagen I, and treated with 10 µM Rhosin, 200 µM Box5 or a mixture of both. Invasion was visualized using microscopy after 48 h. Representative images are shown from 6 independent experiments for both cell lines. The scale bars represent 200 µm.
